# Willingness to Use the Oral Fluid HIV Rapid Test among Men Who Have Sex with Men in Beijing, China

**DOI:** 10.1371/journal.pone.0064652

**Published:** 2013-05-23

**Authors:** Yunan Xu, Zheng Zhang, Dongliang Li, Yingjie Liu, Stephen W. Pan, Xiao Qi, Bo Wang, Fengji Luo, Dong Xiao, Yiming Shao, Yuhua Ruan

**Affiliations:** 1 State Key Laboratory for Infectious Disease Prevention and Control, and National Center for AIDS/STD Control and Prevention (NCAIDS), Chinese Center for Disease Control and Prevention (China CDC), Beijing, China; 2 Chaoyang Center for Disease Control and Prevention, Beijing, China; 3 The University of British Columbia School of Population and Public Health, Vancouver, Canada; 4 Chaoyang Chinese AIDS Volunteer Group, Beijing, China; George Mason University, United States of America

## Abstract

**Background:**

Early detection of HIV infection enables timely care and treatment. However, many men who have sex with men (MSM) remain unaware of their HIV status because they do not or are unable to access HIV testing services. Oral fluid HIV rapid tests have the potential to increase HIV testing. This study is the first to evaluate willingness to use the oral fluid test among MSM in China.

**Methods:**

A cross-sectional study was conducted in Beijing from July to October, 2012. Data were collected by self-administered questionnaires.

**Results:**

Of 262 who participated in the survey, 223(85.1%) reported that they were willing to use the oral fluid HIV rapid test. Willingness to use the oral fluid test was associated with higher education (adjusted odds ratio (AOR): 2.40, 95% confidence interval (CI): 1.13–5.10), lack of unprotected anal intercourse (UAI) with male partners in the past one month (AOR: 2.38; 95% 95%CI: 1.15–4.95), having taken more than 4 HIV tests (AOR: 3.54; 95%CI:1.52–8.28), and having ever heard of the oral fluid HIV rapid test from gay friends or gay organizations (AOR: 3.24, 95%CI: 1.40–7.51). Among those who expressed willingness to use the oral fluid HIV rapid test, the median amount of money they were willing to pay was 8 dollars. Among the 39 participants who were unwilling to use the oral fluid test, 79.5% (31/39) expressed concerns about the accuracy of the oral fluid HIV rapid test results and 17.9%(7/39) reported that they were not familiar with the oral fluid test and did not know how to use such a test.

**Conclusions:**

A high proportion of MSM in Beijing appear to be willing to use the oral fluid HIV rapid test. Appropriate cost and education measures could help improve acceptance of the oral fluid test.

## Introduction

Men who have sex with men (MSM) are affected by the HIV epidemic in many countries and urgently need effective and appropriate prevention approaches. In China, the most-at-risk subgroups have been changing over time, from injection drug users (IDUs) and plasma donors to heterosexual transmission via commercial sex work and sexual transmission among MSM. In recent years, the number of new HIV infections among MSM has rapidly increased. National HIV/AIDS reports in China showed that 29.4% of all new HIV infection cases in 2011were among MSM, up from 12.2% in 2007[1], [2]. HIVprevalence among MSM is particularly high in some large Chinese cities, for example ranging between 5.1–9.9% in Beijing (the capital city in northeastern China) [3]–[5], and 8.5–16.8% in Chongqing, a metropolitan city in southwestern China [6]–[8]. MSM are not only passively impacted by HIV infection, but are also an important driver of the broader HIV epidemic. Due to pressure from society and family, about 17% of MSM in China currently have female spouses. Moreover, about 26% have had female sexual partners in the past six months [9], [10]. MSM who also have sex with women can potentially transmit the virus to the general female population. Therefore, MSM should be considered an important target population of HIV prevention and control.

Recent HIV prevention research has focused on methods to promote frequent HIV testing and awareness of HIV status. Individuals who are aware of their HIV-positive status may be more inclined to modify risky behaviors associated with HIV infection and transmission (e.g., unprotected sex or serodiscordant partner selection) [11], [12]. Moreover, diagnosis of HIV infection is conducive to timely treatment with anti-retroviral therapy (ART), which can suppress viral loads and reduce population-level HIV transmission [13]–[15]. Such prevention approaches are heavily dependent on participation in HIV testing and awareness of HIV status. Therefore, HIV testing is not only an entry point for HIV prevention, but also the backbone of innovative combinatorial HIV prevention approaches such as testing, linkage to care and treatment as prevention[16], [17].

In order to improve the rate of HIV testing and encourage awareness of HIV status, the Chinese government has established free HIV testing programs in HIV Voluntary Counseling and Testing (VCT) clinics[18], [19]. Despite government efforts to promote universal access to HIV testing, many HIV-infected individuals remain unaware of their HIV status; it is estimated that up to 56% of the 780,000 individuals living with HIV/AIDS in mainland China in 2011 were unaware of their HIV status [2]. A recent survey in Beijing City found that 33.2% of MSM had never taken an HIV test and 86.1% of HIV-positive MSM were unaware of their infection [20]. One reason why individuals do not test for HIV is because of perceived discomfort, pain and fear of blood that comes with traditional blood-based tests [21]. Furthermore, some individuals who are tested may not be aware of their HIV positive status because they do not return to the clinic for their test results[22]. The relatively high prevalence of infrequent testing and unrecognized HIV infection presents a formidable barrier to the scale-up of HIV testing and prevention, and suggests that changes to the current HIV testing service model may help increase HIV testing among MSM. One such approach is the oral fluid HIV rapid test, which has several notable advantages over traditional HIV tests. Compared with blood-based tests, the oral fluid tests are relatively convenient, noninvasiveness, expedient, and has high cultural acceptance among disparate racial/ethnic groups, while maintaining a high degree of accuracy [23]–[26]. Such features of the oral fluid test can help improve the rates of HIV testing, without compromising testing accuracy.

However, to our knowledge, no studies have focused on evaluating willingness to use the oral fluid HIV rapid test in China. To assess the feasibility of using the oral fluid HIV testing among MSM in China, this study seeks to assess the willingness to uptake the oral fluid HIV rapid test and its associated factors among MSM in China.

## Methods

### Study design and participants

This cross-sectional study was conducted among MSM at a VCT site at the Beijing Jingcheng Skin Disease Hospital from July to October 2012. The participants were recruited in four ways. First, participants were recruited through advertisements on a website of a nongovernmental AIDS volunteer organization that provides information about HIV testing and counseling (www.hivolunt.net). Second, two peer recruiters were hired and trained to call in people who had ever participated in similar programs before. Third, people who came to the clinic for regular HIV testing or counseling voluntarily were also encouraged to participate in the study. Fourth, subjects were encouraged to introduce their gay friends to participate in the study. Participants were eligible if they self-reported: having had sex with men in the past 6 months, being 18 years or older, unaware of any HIV infection, and willing to participate and able provide written informed consent. After obtaining written informed consent, trained interviewers provided a brief introduction about HIV testing methods, including a description about the enzyme-linked immunosorbent assay (ELISA), blood-based rapid HIV test and the oral fluid rapid test. Then, previously-validated questionnaires were self-administered by participants. After the questionnaires were completed, confidential HIV pre-test counseling and the traditional blood-based HIV rapid test was conducted. Finally, interviewers informed the participant of their test results and provided post-test counseling according to the results. The study protocol and informed consent form were approved by the institutional review board of the National Center for AIDS/STD Control and Prevention of the Chinese Center for Disease Control and Prevention.

### Laboratory tests

HIV infection status was screened for HIV-1 antibodies by blood-based rapid test(Determine HIV-1/2, Abbott Japan Co., Japan) and confirmed by HIV-1/2 Western Blot (HIV Blot 2.2 WBTM, Genelabs Diagnostics, Singapore). Syphilis screening was performed by rapid plasma reagin(RPR; Shanghai Rongsheng, Shanghai, China) and confirmed by Passive Particle Agglutination Test for Detection of Antibodies to Treponema pallidum (TPPA™, FUJIREBIO, Japan).

### Data collection

Data were collected by participant self-administered questionnaires completed in a private room. The contents of the structured questionnaire included willingness to use the oral fluid rapid HIV test, socio-demographic characteristics (age, ethnicity, education level, marital status, cohabitation status, employment status, has permanent Beijing residency status, years living in Beijing, monthly income and health insurance), sexual behaviors(age at the first insertive sexual intercourse with male, sexual orientation, role in anal sex with men, the number of male sexual partners in the past 6 months, unprotected anal intercourse (UAI) with males and unprotected vaginal sex with female partners in the past 1 month), sexually transmitted diseases (STDs), HIV testing experiences (ever having taken a free or self-paid HIV ELISA test or rapid test, the lifetime number of times having taken an HIV testing, having taken an HIV test in the past one year, knowledge that both hospitals and the CDC can provide HIV tests, having ever heard of the oral fluid rapid test, having ever heard of the oral fluid HIV rapid test from gay friends or gay organizations, perceived accuracy of oral fluid HIV rapid test results), and HIV and Syphilis test results.

Participants who expressed willingness to use the oral fluid tests were asked how much they were willing to personally pay for such a test. Participants who were unwilling to use the oral fluid test were asked to briefly explain in an open ended response why they were unwilling to use the oral fluid test.

### Data analysis

Collected data were double-entered and compared using EpiData3.1 (The EpiData Association Odense, Denmark). After cleaning, the data were converted and analyzed with Statistical Analysis System (SAS 9.2 for Windows; SAS Institute Inc, NC, USA). Univariate logistic regression analysis was performed to evaluate associations of willingness to use oral fluid rapid tests with socio-demographic characteristics, sexual behaviors, sexual health status, and HIV testing. Odds ratios(OR) and 95% confidence intervals(95%CI) were calculated accordingly. Variables with a P-value <0.10 in the univariate analysis were eligible for entry into the multivariate logistic regression model, where adjusted OR (AOR) and 95%CI were calculated accordingly. A mixed stepwise approach was used to build the final multivariate logistic regression model. Statistical significant was defined as P<0.05 (two-tailed test).

## Results

### General characteristics

A total of 288 participants were screened for the study. Nineteen reported that they did not have sex with men in the past 6 months and 7 already knew their HIV positive status. Therefore, 262 MSM were included in the analysis. The median age was 29 years (interquartile range: 25–33) with a range from 19 to 63. Most participants (93.5%) belonged to the Han ethnic group; about half (50.76%) of participants completed college or higher levels of education; 38.9% earned more than $800USD per month. Among these variables, only education level was marginally associated with willingness to use the oral fluid rapid test in the univariate regression model (table 1).

**Table 1:** Associations between willingness to use the oral fluid HIV rapid test and socio-demographic characteristics, among Men who have sex with men in Beijing, China.

Characteristic	Willingness to use the oral fluid HIV rapid test		OR	95%CI	P-value
	event/total	%			
Age(years)					
≤25	55/68	80.9	1.0		
>25	168/194	86.6	1.53	0.73–3.18	0.26
Ethnicity					
Han	209/245	85.3	1.0		
Others	14/17	82.4	0.80	0.22–2.94	0.74
Education					
High school or lower	105/129	81.4	1.0		
College or higher	118/133	88.7	1.80	0.90–3.61	0.10
Current marital status					
Unmarried	187/217	86.2	1.0		
Married	36/45	80.0	0.64	0.28–1.47	0.29
Cohabitation status					
Live alone	132/154	85.7	1.0		
Cohabitation with others	91/108	84.3	0.89	0.45–1.77	0.74
Employment status					
Employed	196/229	85.6	1.0		
Unemployed	27/33	81.8	0.76	0.29–1.98	0.57
Has permanent Beijing residency status					
Yes	64/75	85.3	1.0		
No	159/187	85.0	0.98	0.46–2.08	0.95
Years living in Beijing					
≤2	31/40	77.5	1.0		
>2	192/222	86.5	1.86	0.81–4.29	0.15
Monthly income(USD) in the past one year					
≤800	134/160	83.8	1.0		
>800	89/102	87.3	1.33	0.65–2.72	0.44
Has health insurance					
Yes	154/176	87.5	1.0		
No	69/86	80.2	0.58	0.29–1.16	0.12

OR, odds ratio; 95%.

95%CI, 95% confidence interval.

Most participants (82.4%) reported being fewer than 26 years old at the time of their MSM sexual debut. Slightly less than half of participants (43.5%) had >2 male sexual partners in the past 6 months. Prevalence of unprotected anal sex with male partners in the past 1 month and any unprotected sex with female partners in the past 1 month was 30.5% and 6.1%, respectively. About four fifths (81.7%) of participants reported that they had ever received an HIV test and 69.9% had ever received an HIV rapid test. Overall, 58.0% reported having received an HIV test more than 4 times in their life. 40.1% heard of the oral fluid HIV rapid test from gay friends or gay organizations and 65.7% trusted results of the oral fluid test. 6.9% were confirmed to be infected with HIV and 21.4% with Syphilis. Regarding measures of association with willingness to use the Oral HIV test, there were ten variables that had P-values below 0.10 in the univariate regression models (table 2).

**Table 2:** Associations between willingness to use oral fluid HIV rapid test and sexual behaviors and, HIV testing among Men who have sex with men in Beijing, China.

Characteristic	Willingness to use the HIV oral fluid rapid test		OR	95%CI	P-Value
	event/total	%			
Age at the first insertive sexual intercourse with male (years)					
≤25	189/216	87.5	1.0		
>25	34/46	73.9	0.41	0.19–0.88	0.02
Self-reported sexual orientation					
Homosexual	168/194	86.6	1.0		
Bisexual or uncertain	55/68	80.9	0.66	0.32–1.36	0.26
Self-reported role in anal sex with men					
Exclusive insertive anal sex	47/59	79.7	1.0		
Exclusive receptive anal sex	16/20	80.0	1.02	0.29–3.62	0.97
Both	160/183	87.4	1.78	0.82–3.84	0.14
The number of male sexual partners in the past 6 months					
≤2	131/148	88.5	1.0		
>2	92/114	80.7	0.74	0.52–1.04	0.08
Unprotected anal intercourse with male partners in the past 1 month					
Yes	62/80	77.5	1.0		
No	161/182	88.5	2.23	1.11–4.46	0.02
Unprotected sex with female partners in the past 1 month					
Yes	11/16	68.8	1.0		
No	212/246	86.2	1.83	0.93–8.66	0.07
Diagnosed with STD in the past one year					
Yes	41/53	77.4	1.0		
No	182/209	87.1	1.97	0.92–4.22	0.08
Has ever taken an HIV test					
Yes	181/214	84.6	1.0		
No	42/48	87.5	1.28	0.50–3.24	0.61
Has ever taken a free HIV test					
Yes	175/208	84.1	1.0		
No	48/54	88.9	1.51	0.60–3.81	0.38
Has ever paid for an HIV test					
Yes	62/69	89.9	1.0		
No	161/193	83.4	0.57	0.24–1.35	0.20
Lifetime total number of HIV tests ever taken					
≤4	121/152	79.6	1.0		
>4	102/110	92.7	3.27	1.44–7.42	<0.01
Has taken an HIV test in the past one year					
Yes	148/174	85.1	1.0		
No	75/88	85.2	1.01	0.49–2.09	0.97
Has ever taken an HIV rapid test					
Yes	156/183	85.3	1.0		
No	67/79	84.8	0.97	0.46–2.02	0.93
Has ever taken a free HIV rapid test					
Yes	154/181	85.1	1.0		
No	69/81	85.2	1.01	0.48–2.11	0.98
Has ever paid for an HIV rapid test					
Yes	25/28	89.3	1.0		
No	198/234	84.6	0.66	0.19–2.30	0.51
Knows that hospitals and the CDC both offer HIV testing					
Yes	127/141	90.1	1.0		
No	96/121	79.3	0.42	0.21–0.86	0.02
Has ever heard of the oral fluid HIV rapid test					
No	51/67	76.1	1.0		
Yes	172/195	88.2	2.35	1.15–4.77	0.02
Has ever heard about the oral fluid HIV rapid test from gay friends or gay organizations					
No	127/157	80.9	1.0		
Yes	96/105	91.4	2.52	1.14–5.56	0.02
Trusts in the accuracy of the oral fluid HIV rapid test					
Yes	151/172	87.8	1.0		
No or uncertain	72/90	80.0	0.56	0.28–1.11	0.10
HIV serostatus					
Negative	207/244	84.8	1.0		
Positive	16/18	88.9	1.43	0.32–6.47	0.64
Syphilis serostatus					
Negative	172/206	83.5	1.0		
Positive	51/56	91.1	2.02	0.75–5.42	0.16

OR, odds ratio;

95% CI, 95% confidence interval.

### Predictors of willingness to use the oral fluid HIV rapid test

Of 262 survey participants, 85.1% (223) reported that they were willing to use the oral fluid HIV rapid test. A total of eleven variables were statistically significant at the level of 0.10 in univariate analysis and therefore were included in a multivariate logistic regression model. Independent predictors of willingness to use the oral fluid test were higher education (AOR: 2.40, 95%CI: 1.13–5.10), lack of UAI with male partners in the past 1 month (AOR: 2.38; 95% 95%CI: 1.15–4.95), having taken more than 4 HIV tests (AOR: 3.54; 95%CI: 1.52–8.28), and having ever heard of the oral fluid HIV rapid test from gay friends or gay organizations (AOR: 3.24, 95%CI: 1.40–7.51) (table 3).

**Table 3:** Multivariate logistic regression analysis of factors associated with willingness to use oral fluid HIV rapid test, among Men who have sex with men in Beijing, China.

Characteristic	AOR	95%CI	P-value
Education			
High school or lower	1.0		
College or higher	2.40	1.13–5.10	0.02
Unprotected anal intercourse with male partners in the past 1 month			
Yes	1.0		
No	2.38	1.15–4.95	0.02
Lifetime total number of HIV tests ever taken			
≤4	1.0		
>4	3.54	1.52–8.28	<0.01
Has ever heard about the oral fluid HIV rapid test from gay friends or gay organizations			
No	1.0		
Yes	3.24	1.40–7.51	<0.01

AOR, adjusted odds ratio;

95% CI, 95% confidence interval.

### Price willing to pay for the oral fluid HIV rapid test and the reasons for unwillingness

85.1% (223/262) participants who reported willingness to use the oral fluid HIV rapid test were asked the cost they willing to pay. The median cost was $8 (interquartile range: $3–$16) with a range from 0 to $80 (table 4). Of 14.9% (39/262) unwilling to use the oral fluid rapid HIV test, 79.5% (31/39) expressed concerns about accuracy about result of the oral fluid HIV rapid test and 17.9% (7/39) reported that they were not familiar with it and did not know how to use such test. 2.6% (1/39) thought that it was unhygienic to put an unsterile stick into one's mouth.

**Table 4:** Maximum amount willing to pay for the oral fluid HIV rapid test (RMB∶USD = 6.2∶1).

Price(USD)	n	%
total	233	100
≤1.5	38	17.04
∼3	23	10.31
∼5	15	6.73
∼8	63	28.25
∼16	60	26.91
∼24	8	3.59
∼32	3	1.35
>32	13	5.82

## Discussion

Our study showed that 85.1% participants were willing to use the oral fluid HIV rapid test. Similar to results of many literatures, most MSM and the general population are likely to use the oral fluid rapid test in lieu of traditional blood-based tests. Previous studies performed among risky populations have shown that 64.5%–87.0% of participants prefer the oral fluid-based test over the blood-based test [21], [27], [28]. Favorability towards the oral fluid HIV rapid test may be attributed to its relative convenience and non-invasive nature.

Oral fluid rapid tests also represent a promising opportunity to reduce incidents of missed HIV post-test counseling because tests results are made available within 30 minutes at the testing site or in the participant's own home. In a previous study, nearly half of individuals who received traditional blood-based tests, the results of which are not available until after several days, did not receive post-test counseling [29]. On the other hand, another study showed that the post-test counseling rate for participants who tested with the oral fluid rapid test was 89% for HIV-infected and 93% for uninfected participants [28].

Lack of information about available HIV testing sites represents a challenging barrier to HIV testing among MSM. In one study of MSM in Peru, 31.3% of high-risk MSM reported lack of knowledge regarding where to get tested as a reason for not getting tested for HIV [30]. The oral fluid rapid test can potentially reduce this issue by allowing individuals to assess their HIV status in a flexible variety of non-clinical settings, such as their own home. In a study among internet-using MSM, 82% of participants expressed being very likely or somewhat likely to take a home HIV test [31]. One possible reason for the preference of home testing is because individuals may be disinclined to make two trips to an HIV testing site, once for the test and again for the test result. Therefore, the oral fluid HIV rapid tests represent an appealing and effective way to improve rates of HIV screening and post-test counseling.

According to the results of this study, attention should be paid to two subgroup populations among MSM: MSM with lower education and MSM who engaged in UAI. One study showed that those who have taken an HIV test and knew their results are more likely to have a higher level of education[32]. UAI is not only a primary cause of HIV transmission, but has also been shown to be connected with HIV testing: MSM with UAI in the past year was associated with not having a recent HIV test, and MSM with more unprotected sex had lower intentions of being tested [33], [34]. This implies that MSM with higher levels of education and less UAI may be more efficient at utilizing HIV-testing services and thus detecting personal HIV-positive status at an earlier stage. Meanwhile, results also suggest that those with less education and UAI should be regarded as a priority group for HIV testing outreach. In our study, MSM with college-education and without UAI were more willing to use the oral fluid rapid test. According to study results, concern about testing accuracy and unfamiliarity of oral fluid rapid test are the main reasons for unwillingness to use such a test. Thus one possible explanation may be that MSM with less education and UAI are more concerned about accuracy of the oral fluid test and may have a poorer understanding about it. Therefore, when we promote the oral fluid HIV rapid tests, the two subgroup populations among MSM should be key groups to target.

In our study, having taken more HIV tests and receiving oral fluid test information from gay friends or gay organizations were significantly associated with willingness to use the oral fluid rapid test. Thus, this suggests that education about the oral fluid HIV rapid test may increase willingness to use the test among target groups. The association between willingness to use the oral fluid rapid test and number of HIV tests ever taken may be explained by greater exposure to information about the oral fluid test in VCT clinics. While receiving services at a VCT clinic, participants are exposed to informational flyers and posters about HIV tests and AIDS prevention, and have greater opportunities to familiarize themselves with different tests, such as the oral fluid rapid HIV test. Moreover, the repeated experience traveling to and from their home and the testing site may make the at-home convenience of the oral fluid HIV rapid test particularly appealing.

Peer-based interventions represent a promising avenue for implementing HIV interventions. Peer-based education has been shown to raise the age of sexual debut, increase the use of condoms, improve quality of life and reduce high-risk behaviors [35]–[37]. One study also reported that a substantial proportion of clients were willing to receive peer-delivered HIV test and counseling [38]. It was encouraging to note that participants who heard of the oral fluid HIV rapid test from gay friends or gay organizations were more willing to use the oral fluid rapid HIV tests, suggesting that that MSM may be more motivated by HIV information from members of their own communities. Peer-based education may be an effective means of promoting adoption of the oral fluid HIV test.

Currently, there is no government financial support for subsidizing oral fluid rapid HIV tests in China, and consumers must pay for the oral fluid tests out-of-pocket. Excessively high prices may discourage adoption of the oral fluid test in China, despite its obvious advantages. Our study reported that more than 60% of participants were willing to pay up to$8, while very few (10.8%) were willing to pay the current market price of the oral fluid rapid test in China, which is currently more than $16. It is also possible that low-income populations from poorer areas, which often have a greater HIV burden, may be even more sensitive to pricing than participants from Beijing. Therefore, the price of oral fluid rapid test must be set within the range MSM/consumers are willing to pay if the oral fluid test is used to scale up HIV screening.

Almost 15% of participants were not willing to use an oral fluid HIV rapid test. Among the reasons why individuals were not willing to use the oral fluid test, concern about testing accuracy was most common, followed by unfamiliarity with such a test. Similar to all other tests, false-negative, false-positive and nonreactive results are also possible with the oral fluid rapid HIV test. But overall, the oral fluid test has about the same accuracy as blood-based tests and has appeared to work well among MSM in resource-limited settings in India [21], [26], [39]. In light of successful roll-out of the oral fluid test among MSM in other countries, successful scale-up of the oral fluid rapid test in China is promising. From a public health research perspective, the result is important for providing some of the most common and main reasons given for unwillingness to use oral fluid rapid test. Efforts to alleviate concerns about accuracy and ignorance may lead to higher levels of HIV testing using the oral fluid HIV rapid test among MSM in China.

This study has several limitations that should be noted. First, a convenience sample may have resulted in selection bias. Non-participants may have had different characteristics in terms of socio-demographics, sexual behaviors, HIV testing experience and willingness to use the oral fluid test. The sample also may not be representative of MSM in Beijing or China. Because of Beijing residents have a relatively high socio economic status relative to the rest of China, it can be inferred that people outside of Beijing may be more likely in need of a convenient and effective HIV test. Second, this investigation addressed sensitive questions which may have led to misreporting of personal attitudes and behaviors due to social desirability bias. Participants may have underreported the extent of their risk sexual behaviors or over-reported the frequency of their HIV tests. Third, this study only assessed willingness to use the oral fluid test, rather than actual adoption of the oral fluid test. Therefore, further research is needed to explore the adoption of the oral fluid tests in actual practice.

In summary, despite these limitations, our study has important implications for scaling-up of the oral fluid HIV rapid testing among MSM in China. The high rate of willingness to use the oral fluid test indicates that promotion of the oral fluid rapid test may be an effective way to improve rates of HIV screening and counseling among MSM in China. Peer education by other MSM may be an effective mean of promoting the oral fluid HIV rapid tests, and should emphasize the accuracy of the tests, relative to standard blood-based tests. Promotion of the oral fluid rapid testing should focus on MSM with lower-levels of education and higher risk behaviors. It is important to ensure that the oral fluid test is appropriately priced in resource-limited areas where people need them most. Meanwhile, further research should be done to assess the feasibility of using the oral fluid rapid test in resource-limited areas and rural settings among risky population in China.
