# Correction: Willingness to Use the Oral Fluid HIV Rapid Test among Men Who Have Sex with Men in Beijing, China

**DOI:** 10.1371/annotation/47ef6213-e854-4dca-b321-704c261c6167

**Published:** 2014-01-06

**Authors:** Yunan Xu, Zheng Zhang, Dongliang Li, Yingjie Liu, Stephen W. Pan, Xiao Qi, Bo Wang, Fengji Luo, Dong Xiao, Yiming Shao, Yuhua Ruan

The number of an additional grant from the Ministry of Science and Technology of China was incorrectly omitted from the Funding Statement. The grant number is: 2012ZX10004-904. 

